# Comparison of animal welfare assessment tools and methodologies: need for an effective approach for captive elephants in Asia

**DOI:** 10.3389/fvets.2024.1370909

**Published:** 2024-03-12

**Authors:** Raman Ghimire, Janine L. Brown, Chatchote Thitaram, Pakkanut Bansiddhi

**Affiliations:** ^1^Faculty of Veterinary Medicine, Chiang Mai University, Chiang Mai, Thailand; ^2^Center of Elephant and Wildlife Health, Chiang Mai University Animal Hospital, Chiang Mai, Thailand; ^3^Elephant, Wildlife, and Companion Animals Research Group, Chiang Mai University, Chiang Mai, Thailand; ^4^Center for Species Survival, Smithsonian Conservation Biology Institute, Front Royal, VA, United States

**Keywords:** animal welfare, captive elephant, welfare assessment, framework, tourist camp, zoo

## Abstract

Welfare is a fundamental aspect of animal management and conservation. In light of growing public awareness and welfare concerns about captive elephants, there is an urgent need for comprehensive, globally coordinated efforts for Asian elephants *(Elephas maximus)* that participate in religious, logging, or tourist activities in range countries where the majority reside, and where welfare issues have been identified but not addressed. This review provides a comparative analysis of available animal assessment tools. Each offers distinct features for assessment that allow institutions to select criteria for specific needs and available resources. Most are applied to general animal welfare assessments, although some are tailored to particular species, including elephants. The tools span diverse formats, from digital to primarily paper-based assessments. Assessments operate at individual and institutional levels and across multiple welfare domains. Methodologies rely on keeper ratings or expert evaluations, incorporate numerical scoring and Likert scales for welfare grading, and encompass inputs including behaviors, health, and physiological indicators. For tourist camp elephants, one challenge is that the tools were developed in zoos, which may or may not have application to non-zoological settings. Digital tools and assessment methodologies such as keeper ratings face logistical challenges when applied across tourist venues. As with any tool, reliability, validity, and repeatability are essential and must address the unique welfare challenges of diverse captive settings. We propose that a holistic, context-specific, evidence-based, and practical tool be developed to ensure high elephant welfare standards in non-zoological facilities throughout Asia.

## 1 Introduction

Animal welfare is a multifaceted concept that increasingly focuses on the cumulative physical, psychological, and behavioral states of individual animals (1). It encompasses scientific, ethical, economic, cultural, and religious dimensions with varying perspectives among scholars (2, 3). Initially, animal welfare science focused on enhancing the welfare of production and laboratory animals (4, 5). The Farm Animal Welfare Advisory Committee (FAWAC) took a significant step in 1965, developing the Five Freedoms model to address farm animal welfare concerns (6). It stated that animals should be free from hunger, discomfort, pain, and fear, and able to express natural behaviors. The model dominated discussions on animal welfare in Europe for decades (7), serving as a comprehensive framework while acknowledging the operational constraints of the livestock industry (8). However, criticisms surfaced, questioning its practicality and minimal emphasis on positive welfare experiences (9), prompting the development of alternative frameworks. The Five Domains Model offers a holistic approach focusing on affective terms and recognizing the subjectivity in measuring mental experiences (10). This emphasis on mental well-being aligns with broader ethical, policy, and legal considerations in contemporary animal welfare science. The model integrates the concept of agency within Domain 4 (Behavioral Interactions), enabling the evaluation of animal engagement in voluntary, self-generated, and goal-directed behavior (11) and human-animal interactions (10). Widely accepted in farm and zoo communities, the Five Domains Model has been adopted by organizations like WAZA (12) and the Zoo and Aquarium Association, Australasia (13) to uphold high welfare standards. However, to effectively utilize the model as a welfare assessment tool, attention should be given to using well-validated measures, ensuring transparency in expert panel selection, and implementing a clear welfare grading system (14).

Numerous welfare assessment frameworks have emerged by incorporating the Five Freedoms and Five Domains models. For example, Welfare Quality builds upon the Five Freedoms Model and integrates scientific expertise and ethical considerations from various stakeholders, including the general public, industry, and political bodies, to evaluate welfare (4). It prioritizes animal-based measures and follows a bottom-up approach, assigning scores based on four crucial principles: proper nourishment, suitable housing, good health, and appropriate behavior. These principles serve as the basis for evaluating overall welfare, and their scores are combined to determine the final assessment. The Opportunity to Thrive Program flips the concept of the Five Freedoms to focus on achieving a positive welfare state, with a particular emphasis on reintegrating animals back into their natural habitats (15). The framework offers a comprehensive method for managing animals, incorporating formulated diets, environmental design, healthcare, enrichments, choice and control, and access to species-typical behavior. These inputs ultimately aim to achieve desired outputs, resulting in an overall animal welfare assessment. A 24/7 approach was proposed to evaluate zoo animal welfare, utilizing the 12 welfare assessment criteria from the Welfare Quality framework (16). This approach considers the natural history, biology, ecology, diet, habitat, social structure, and activity patterns of animals throughout both day and night, providing a thorough understanding of their welfare. Finally, the Universal Animal Welfare Framework is an institutional-level welfare assessment framework based on the Five Domains Model (17). Developed by the Detroit Zoological Society in 2015, it examines zoo practices, policies, resources, and measures related to housing, routine, and behavior.

Both species-specific and species-general welfare assessment tools have utilized these welfare models and frameworks. Generalized tools work under the assumption that animals have the same basic needs, so management should be based on natural history. However, these tools face challenges in addressing species-specific nuances. A few have been developed for specific species and include the giant pacific octopus (*Enteroctopus dofleini*) (18), pygmy blue-tongued skink (*Tiliqua adelaidensis*) (19), bottle-nosed dolphin (*Tursiops truncates*) (20), waterfowl (Anseriformes) (21), dorcas gazelle (*Gazella dorcas*) (22), and elephant (*Elephas maximus*) (23).

These offer a refined and more precise evaluation of animal welfare by tailoring assessments to the unique needs, behaviors, and physiological aspects of a particular species (24). For some species with special spatial, environmental, social, or cognitive needs, a “one-size-fits-all” strategy to assess welfare may not be appropriate; rather, species-specific (25, 26) and if possible, context-specific assessment tools are needed.

Elephants, characterized by their large body size, complex social lives, varied food requirements, and extensive wild home ranges (27–29), pose challenges to meeting physical, psychological, and physiological needs in human-created environments (30). Ensuring good welfare for these animals involves allowing some degree of choice and control. Additionally, wild elephants spend about 80% of the time foraging and are highly social (31). Denying these freedoms can result in maladaptation, chronic stress, poor welfare (32, 33), and abnormal stereotypic behaviors (34, 35). Good zoos provide health care, safety from predation, and food security (30) and aim to meet exercise (36), foraging, and social complexity (37) needs. However, high mortality, low birth rates, limited reproduction, and health problems continue to hamper zoo elephant population sustainability (38–42), igniting worldwide concerns over animal welfare (43, 44). In 2016, a series of epidemiological studies of elephants in North American zoos revealed problems associated with ovarian acyclicity (45), health and musculoskeletal function (46), stereotypic behaviors (35), and high body condition scores (47). A similar set of studies on tourist elephants in Thailand found similar problems associated with elevated stress hormones (48), excessive body condition and metabolic derangements (49), and stereotypies (50). Finally, surveys of thousands of elephants in hundreds of tourist venues across Thailand, India, Nepal, Sri Lanka, Laos, Cambodia, and Malaysia suggest that more than half (63%) are kept in inadequate conditions (51–53). There is little doubt that comparable situations exist for logging (54), temple (55, 56), and circus (57) elephants as well. Thus, while the problematic state of captive elephant welfare across diverse conditions is now well-known, the solutions have proven far more elusive.

Considerable attention has been directed toward improving zoo elephant welfare, while the unique conditions and challenges faced by captive elephants in non-zoo settings are often overlooked (58). There are over 14,000 captive Asian elephants outside traditional zoo environments across 13 range countries, primarily in tourist or logging camps and temples (58). There are notable differences in the management of zoo and camp elephants. Zoo elephants are typically managed in protected contact systems, minimizing direct interaction with humans and other practices that adhere to standardized regulations (59). Staff are responsible for feeding, bathing, training, and veterinary care; however, because of limited space, socialization and exploration can be limited. In contrast, camp elephants are often managed in free contact where elephants and people share the same space, including with tourists (60). Daily routines involve tourist-related tasks such as shows, riding, walking, bathing, feeding, or observation (60). Some elephants participate in cultural activities like religious rituals and festivals (61). Welfare can be better in camps situated in natural environments, with forests and rivers providing more natural foraging and exercise opportunities than zoos (62). However, restraint methods like chaining and using an ankus (also known as a bullhook or guide) to control elephants are significant concerns (63). In addition, the lack of enforceable standards results in varied management practices across and even within camps (64), which ultimately poses challenges in addressing the welfare needs of camp elephants. Animal activists continually voice concerns regarding the welfare and management of tourist camp elephants (65, 66). Thus, there is a need for a holistic, evidence-based welfare assessment approach to identify potential welfare risks, inform management decisions, and record welfare changes over time (67, 68). It also can contribute to elephant welfare standardization and policy-making processes crucial for properly managing elephants in range countries.

This review examines available generic and elephant-specific welfare assessment tools and methodologies and discusses applicability to tourist camp elephants (Tables 1, 2). While there have been several reviews of animal welfare frameworks (24–26), to our knowledge, this is the first overview of welfare assessment tools specific to elephants. Predefined criteria guided the selection of welfare assessment tools for this review to ensure a representative and comprehensive overview. Those included relevance to captive elephant welfare, recognition and adoption in the scientific community, and diversity of approaches. The featured tools were carefully chosen to provide readers with meaningful insights into the diversity and applicability of current welfare assessment practices for captive elephants, acknowledging that the selection may not encompass every existing tool. Our ultimate goal is to synthesize a new welfare assessment tool specific to elephants used in tourism, considering the strengths and limitations of existing tools and challenges faced by tourist camps.

**Table 1:** Summary of available welfare assessment tools.

Tool	Developer	Online or paper-based	Assessment level	Measures used	Assessment methodologies
ZooMonitor	Lincoln Park Zoo	Online	Individual	Behavioral activity budget and diversity, space use	Observations using camera traps, CCTV footage, or in-person observations
WelfareTrak	Chicago Zoological Society (CZS)	Paper-based	Individual	Ten animal-based measures including physical health and behavioral indicators	Keeper-based ratings using 5-point Likert scale
Zoological Information Management System (ZIMS) for Care and Welfare	Species360	Online	Individual and Institutional	Based on the Five Domains model: Nutrition Environment Health Behavior Mental health	Information gathering and sharing application Users select indicators and grading scales for welfare assessments based on species requirements
Welfare Discussion Tool (WDT)	Lincoln Park Zoo	Online	Individual and Institutional	41 resource and animal-based welfare measures	4-point scale (2 strongly disagree; 1 moderately disagree; +1 moderately agree; +2 strongly agree) Assessments conducted by: Curator or manager Caretakers Animal experts
Animal Welfare Assessment Grid (AWAG)	Wolfensohn et al. (69)	Online	Individual	Modified Five Domains model: Physical Psychological Environmental Medical procedures	Keeper-based rating using a 10-point numerical scale
Animal Welfare Risk Assessment Process (AWRAP)	Sherwen et al. (67)	Paper-based	Institutional	Modified Five Domains model: Environment (physical/social) Behavior Physical health/nutrition Husbandry	Keeper-based rating using a scale of 0 (highest overall welfare risk) to 2 (lowest overall welfare risk)
Ackonc-Animal Welfare Assessment (AWA)	Racciatti et al. (70)	Paper-based	Individual and Institutional	Modified Five Domains model: Nutrition Environment Health Behavior / mental state	Keeper-based rating using a 3-point scale (A - normal/no observable welfare risk; B - mild deviation/welfare risk; C - Severe deviation/welfare risk)
Wild Welfare Animal Welfare Collection Assessment	Wild Welfare	Paper-based	Individual and Institutional	Based on the Five Domains model: Nutrition Environment Health Behavior Mental health	Expert-based measures are scored as Unacceptable Questionable Acceptable
Elephant Behavioral Welfare Assessment Tool (EBWAT)	Elephant Welfare Project under the British and Irish Association of Zoos and Aquariums (BIAZA)	Online and Paper-based	Individual	Qualitative Behavioural Assessment (QBA) and Behavioral Ethogram containing daytime and nighttime activity	Keeper-based rating using a Likert scale with responses ranging from ‘never’ to ‘more than once per day’ where appropriate and utilized various numbers of response options based on the expected frequency of that behavior
Elephant Welfare Initiative (EWI)	Association of Zoo and Aquariums (AZA) Elephant Taxon Advisory Group	Online	Individual and Institutional	Based on the findings of multi-institutional epidemiological studies conducted in North America Resource-based measures (inputs) include housing features and management practices; animal-based measures (outputs) include behavior and physical health	Resource-based measures presented as logos indicating how goals were met during the day (sun logo), during the night (moon logo), or both Values indicate the percentage of each behavior observed Body condition score based on Morfeld et al. (47) Data based on direct observation by EWI members (experts)
Captive Elephant Welfare Index	Gurusamy and Phillips (71)	Paper-based	Individual	Factors include enclosure substrate, group size, health care, enrichment, restraining the animal, enclosure type, exercise provision, enclosure size, interaction with keeper and training, enclosure environment, keeper knowledge and experiences, diet, keeper contact method, display duration, and enclosure security	Expert-based rating using different scales; e.g., group size (1–4), display duration (1–5), and exercise provision (1–6)
World Animal Protection (WAP) Assessment	Schmidt-Burbach et al. (51–53)	Paper-based	Institutional	Based on Five Freedoms and Welfare Quality Factors include mobility, hygiene and shelter, environmental noise quality, the naturalness of the environment, social interaction, diet, entertainment intensity, and animal management	Expert-based rating using a 5-point scale with 1 being severely inadequate
ABTA Animal Welfare Guidelines: Elephants in Captive Environments	Association of British Travel Agents (ABTA)	Paper-based	Individual and Institutional	Based on Five Freedoms and Welfare Quality 12 criteria under good feeding, good housing, good health, and appropriate behavior domain of Welfare Quality along with three additional criteria addressing animals in tourism	Factors are divided into bad or best practices
Guidelines on the Usage of Captive Elephants in Malaysia	Malaysian Association of Zoological Parks and Aquaria (MAZPA)	Paper-based	Individual and Institutional	Guidelines include better housing and care, no physical abuse, provision of positive reinforcement, and others	No specific scoring system Body condition is scored using a scale; 0–5 = emaciated, 6–10 = average and > 10 = fat or very good condition

**Table 2:** Strengths and limitations of available welfare assessment tools.

Tool	Strengths	Limitations or Challenges (focused on tourist camps)
ZooMonitor	Continuously updated across platforms, including iOS, Android, and Windows devices Flexible for in-person observation or CCTV footage Allows 24/7 systematic behavioral and social interaction monitoring	Relies on behavioral observations that may be too time-consuming for mahouts Mahouts may have limited knowledge of elephant biology and behavior for proper assessment Integration of husbandry records required for holistic welfare assessment requires expertise Challenges in low-budget venues and non-English-speaking regions
WelfareTrak	Quantitative scoring and flagging systems for setting standards and tracking alterations over time play a crucial role	Integration of resource-based measures is necessary for holistic assessment and may be lacking The subjective nature of mahout assessments may introduce bias
Zoological Information Management System (ZIMS) for Care and Welfare	Holistic approach to welfare assessment using animal and resource-based measures Facilitates global sharing of information and data storage Allows users to specify parameters and select grading scales	Challenges in low-budget venues and non-English-speaking regions Constantly updating information in ZIMS is logistically challenging Implementing ZIMS might reveal welfare issues and require costly improvements that conflict with a camp’s profit-oriented approach, making them hesitant to adopt the system Public disclosure of welfare records may lead to negative publicity affecting the reputation and business of tourist venues
Welfare Discussion Tool (WDT)	Holistic approach to welfare assessment using animal and resource-based measures Inter-rater reliability across three raters Regular post-assessment discussion between raters promotes positive management changes	Endocrinological assessment can be challenging Assessment by three raters regularly is time and resource-intensive for low-budget tourist venues
Animal Welfare Assessment Grid (AWAG)	Holistic approach to welfare assessment using animal and resource-based measures Numerical and visual representation allows welfare changes over time	Scores may not correspond with behavioral observation data, relying heavily on mahout assessments Difficult to access software and requires expertise to present the data in the radar chart
Animal Welfare Risk Assessment Process (AWRAP)	Includes benchmark scores for welfare comparisons Holistic approach to welfare assessment using animal and resource-based measures	Focuses only on institutional-level assessment Predominantly focused on resource-based measures (75%) leading to welfare risk assessment rather than overall welfare assessment Reliance on mahout ratings may introduce bias and subjectivity Measures like safety from predators might not be relevant in the context of tourist camp elephants
Ackonc-Animal Welfare Assessment (AWA)	Holistic approach to welfare assessment using animal and resource-based measures Reliable and valid measures are used	Reliance on mahout ratings may introduce bias and subjectivity Limited evidence on widespread adoption and validation
Wild Welfare Animal Welfare Collection Assessment	Holistic approach to welfare assessment using animal and resource-based measures Includes “non-negotiables” and a pre-intervention audit survey to identify common welfare concerns	Implementation might conflict with tourist venues engaging in practices against Wild Welfare’s “non-negotiables.”
Elephant Behavioral Welfare Assessment Tool (EBWAT)	Use of reliable and valid measures Specific to captive elephants	No evidence of widespread adoption and validation of non-zoological institutions Lacks resource-based measures essential for risk assessment across captive institutions Not intended to compare the welfare of elephants across facilities Feasibility, reliability, and validity tested in UK zoos and may not apply to larger sample sizes or different contexts Relying on 24-h monitoring is impractical in tourist camps
Elephant Welfare Initiative (EWI)	Holistic approach to welfare assessment using animal and resource-based measures Provides real-time analysis at individual and institutional levels Allows benchmarking and monitoring over time	Labor and time-intensive input requirements May require technical expertise for effective implementation
Captive Elephant Welfare Index	Utilizes validated measures	Focuses only on institutional-level assessment
World Animal Protection (WAP) Assessment	Specific focus on tourist camps	Assumption and subjective criteria may influence scoring Lacks integral components such as reliable and valid measures, and recent advances in animal welfare Focuses only on institutional-level assessment
Association of British Travel Agent (ABTA) Animal Welfare Guidelines	Specific to non-zoological institutions such as tourist camps	Lacks integral components such as reliable and valid measures, welfare grading system, and recent advances in animal welfare
Guidelines on the Usage of Captive Elephants in Malaysia	Specific to non-zoological institutions including tourist camps in Malaysia	Lacks integral components such as reliable and valid measures, welfare grading system, and recent advances in animal welfare

### 1.1 Species general welfare assessment tools

#### 1.1.1 ZooMonitor

ZooMonitor was developed by the Lincoln Park Zoo as a simple, software-based online tool to record the behavior and space utilization of individual animals using a digital device (72, 73). The tool is designed to examine activity budgets and behavior diversity across multiple zoo species. It allows the user to upload a map of animal habitats and evaluate space use over time. It facilitates 24-h systematic behavioral and social interaction monitoring and is flexible enough to be used with in-person observations or CCTV footage. The tool is continuously updated across iOS, Android, and Windows platforms. ZooMonitor has been adopted by over 200 institutions (72) and used in pygmy hippos (*Choeropsis liberiensis*) (73), penguins (Spheniscidae) (74, 75), chimpanzees (*Pan troglodytes*) (76), Madagascar giant hognose snakes (*Leioheterodon madagascariensis*) (77), tigers (*Panthera tigris*) (78), elephants (79), Japanese macaques (*Macaca fuscata*) (80), and others.

#### 1.1.2 WelfareTrak

WelfareTrak, designed by the Chicago Zoological Society, is a user-friendly animal-based monitoring tool that relies on weekly keeper assessments of individual welfare (81). The tool is based on the concept that animal keepers are the most familiar with individual animals and can detect subtle behavioral changes. The welfare assessment sheet consists of 10 animal-based measures, including physical health (e.g., body condition), positive (e.g., calm-relaxed), and negative (e.g., self-mutilating) behaviors that are scored on a 5-point Likert scale. The quantitative scoring and flagging system of WelfareTrak allows organizations to set standards for animal care, track alterations over time, and objectively assess the efficiency of management practices and the effects of varied settings. The tool has been used successfully in many species including, but not limited to black rhinos (*Diceros bicornis*) (82), cheetahs (*Acinonyx jubatus*) (83), bears (Ursidae) (84), and western lowland gorillas (*Gorilla gorilla gorilla*) (85).

#### 1.1.3 Zoological Information Management System (ZIMS) for Care and Welfare

ZIMS, managed by Species360 (Minneapolis, MN, United States), is a global database that manages data records for zoo and aquarium members. It is utilized by over 1,300 captive institutions in 102 countries for animal management and conservation (86). In addition to clinical and studbook databases, ZIMS has a module to record data related to animal welfare. The Care and Welfare module within ZIMS utilizes a welfare assessment strategy implemented by WAZA (12) based on the Five Domains Model. With elephants, ZIMS has been used to evaluate female social contexts (87), survivorship (88), and hormone cycle patterns (37). At the taxonomic level, each institution can specify parameters and assign anticipated values or ranges to each indicator within a domain. It offers data storage, record-keeping, and global sharing of life history, species biology, and management records. International recording and sharing of information make multi-institutional studies possible, eliminating the constraints of limited sample size in captive settings.

#### 1.1.4 Welfare Discussion Tool (WDT)

The Lincoln Park Zoo developed the WDT for regular assessments of their collection of animals (89). It includes 41 items containing input (resource-based) and output (animal-based) measures related to behavior, endocrine activity (using non-invasive samples such as feces, swabbing skin in amphibians, etc.), husbandry and management practices, keeper interactions and observations, physical appearance, visitor interactions, and training programs. The measures are quantitatively scored on a 4-point scale (2 strongly disagree; 1 moderately disagree; +1 moderately agree; +2 strongly agree); all items also have an option of IDK (I do not know) and NA (not applicable). In two open-ended questions, raters are asked to recommend three improvements for animal welfare. The WDT assessment is conducted on each individual once per calendar year by three raters: (1) curator or manager, (2) animal caretaker, and (3) animal expert. The raters complete the assessments over 2 weeks and meet for discussion, after which the ratings are entered into the Lincoln Park Zoo’s animal records software. While ZooMonitor has provided systematic behavior observation to gain data-driven insights from built-in graphs and reports, WDT presents a comprehensive assessment approach, inter-rater reliability across three raters, quantitative scoring, and regular discussion between raters on post–assessment period to positive management changes to improve animal welfare.

#### 1.1.5 Animal Welfare Assessment Grid (AWAG)

AWAG was developed for assessing the welfare of primates in research institutions (69, 90) but has since been adapted for birds (91), western lowland gorillas (92), giraffes (*Giraffa camelopardalis*), scimitar-horned oryx (*Oryx dammah*), and large felids (tigers, leopards, and cheetahs) (26). Based on the Five Domains Model, the tool divides welfare measures into four categories: physical, psychological, environmental, and medical, and uses a 10-point scale for quantitative measures. This tool allows individual and group-level assessment and presents the welfare measures as numerical and visual (radial chart) data.

#### 1.1.6 Animal Welfare Risk Assessment Process (AWRAP)

The AWRAP was built on the Universal Welfare Assessment Framework and uses five animal-based and 15 resource-based measures divided into the environment, behavior, physical health/nutrition, and husbandry (67). These measures are scored from 0 (highest overall welfare risk) to 2 (lowest risk) based on keeper assessments. An overall welfare score is calculated for each measure and compared to a threshold score, generated from the distribution of scores across 220 enclosures at three zoos, and a criterion for the lowest 5th percentile value is set. Enclosure values below that limit are designated “at highest risk” with immediate welfare action advised, leading to positive management changes and facility adjustments.

#### 1.1.7 Ackonc-Animal Welfare Assessment (AWA)

Ackonc-AWA is a recently developed multi-species tool based on the Five Domains Model that integrates 23 animal-based measures, 19 resource-based measures, and three management-based measures that fall under five domains: nutrition, environment, health, and behavior/mental state (70). Keepers grade each measure on a 3-point scale (A-normal/no observable welfare risk; B-mild deviation/welfare risk; C-severe deviation/welfare risk). It was developed in Spanish and the name is derived from the native Andean word “ackoncahua”, meaning sentinels. The tool has so far been tested on 14 individuals (10 mammals, two birds, and two reptiles) for reliability, validity, and feasibility.

#### 1.1.8 Wild Welfare Animal Welfare Collection Assessment

Wild Welfare is a UK-registered charity focused on welfare training and assessments, creating global partners, and improving animal welfare legislation (93). They have developed a welfare assessment tool based on the Five Domains Model that is used to conduct facility audits composed of 110 questions related to environment, health, behavior, mental state, caretakers, record keeping, health and safety of staff, and financial responsibility. Each measure is scored by experts in captive management and welfare as (1) unacceptable, (2) questionable, or (3) acceptable to identify the most common welfare concerns. As of 2020, 11 zoos in seven developing nations (Brazil, Egypt, Libya, Indonesia, Thailand, Malaysia, and Vietnam) have completed animal care audits (94). Findings often indicate that animal behavior, positive mental states in animals, and human health and safety are all areas that require assistance. Wild Welfare lists several non-negotiables, stating that facilities must use only positive reinforcement techniques and not restrict animal movements, permit animal demonstrations detrimental to physical or psychological well-being, allow feeding by visitors, or permit unregulated breeding.

### 1.2 Elephant-specific welfare assessment tools

#### 1.2.1 Elephant Behavioral Welfare Assessment Tool (EBWAT)

Among the few elephant-specific welfare assessment tools is EBWAT, which utilizes qualitative assessments of individual daytime and nighttime behavior (23). It was developed as a paper-based tool but is currently available as an Android application. The assessment approach involves qualitative evaluations of elephant behavior based on: (1) rating demeanor on a scale of 1–12 in four sets of 1-min observation periods in a single day; (2) daytime observations of comfort, social interactions, resting, feeding and stereotypic behaviors during four sets of 5-min observations during the day over 3 consecutive days; and (3) reviewing of overnight video footage using 30-min scan sampling. The reliability and validity of the tool were tested on 63 elephants at five UK elephant-holding facilities and are now used by 11 UK and Irish zoological facilities (23).

#### 1.2.2 Elephant Welfare Initiative (EWI)

The EWI is a software-based online tool endorsed by the Elephant Taxon Advisory Group of the AZA as a follow-up to a series of multi-institutional epidemiological studies conducted in North America (95). It uses resource-based measures (inputs), including housing features and management practices, and animal-based measures (outputs) of behavior and physical health. The tool uses a web-based software system that allows users to integrate demographics (age, sex, species), housing plans, 24-h daily monitoring, behavioral and body condition scoring tools, and produces a series of welfare reports. It provides real-time analyses at individual and institutional levels that assist in benchmarking and monitoring changes. However, labor and time-intensive input requirements and inconsistencies in data outputs have limited its use.

#### 1.2.3 Captive Elephant Welfare Index

This tool is based on the concept that captive elephant welfare is related to multiple husbandry parameters (71). Ten elephant experts identified 15 welfare indicators: enclosure substrate, group size, health care, enrichment, restraint, enclosure type, exercise provision, enclosure size, keeper interaction and training, enclosure environment, keeper knowledge and experience, diet, keeper contact method, display duration, and enclosure security (96). Different numerical grading scales (1–6) are used to score each measure, which are combined to obtain a total score. These measures were validated by behavioral and physiological (urinary cortisol) measures in Asian elephants managed at three zoos and three sanctuaries. Elephants with low CEWI scores had higher urinary cortisol and exhibited more stereotypic behaviors.

#### 1.2.4 Assessments by World Animal Protection (WAP)

Welfare assessments based on the Five Freedoms and Welfare Quality models have been conducted on thousands of elephants in tourist venues throughout southeast Asia (Thailand, Nepal, India, Sri Lanka, Cambodia, Laos, and Malaysia) by WAP (51–53). Through direct observations of facilities and interviews with staff, numerical scores are assigned to factors such as animal mobility, hygiene and shelter, environmental noise quality, naturalness of the environment, social interactions, diet, entertainment intensity, and animal management on a 5-point scale. Low scores are assigned if elephants are used for tourist activities like riding, bathing, or feeding, chains are used for restraint, and the mahout carries an ankus. However, those assumptions are subjective, raising questions about their validity without considering how the activities are conducted (97). Rating scores range between 1 and 10 and are calculated as follows: FS = (x/xmax)9 + 1, where FS = final rating score, x = husbandry score, and xmax = maximum achievable husbandry score.

#### 1.2.5 ABTA Animal Welfare Guidelines: Elephants in Captive Environments

The Association of British Travel Agents (ABTA) is among the few accredited organizations that have developed guidelines for non-zoological captive elephant management and care (98). Through extensive multi-stakeholder consultations involving experts, scientists, zoological organizations, and NGOs worldwide, ABTA has formulated comprehensive guidelines to ensure the welfare of elephants engaged in tourism. These guidelines prioritize a holistic approach, aligning with the 12 criteria under the Welfare Quality and Five Freedom frameworks, encompassing feeding, housing, health, and behavior domains. The manual delineates negative (bad) and positive (best) practices, identifying key areas that significantly impact elephant welfare. Practices promoting proper diet, suitable housing conditions, adequate healthcare, minimal chaining, opportunities for social interactions, and controlled public feeding contribute to optimal welfare. Conversely, bad practices, such as inadequate diets, substandard housing, insufficient healthcare, excessive chaining, intensive tourist activities, and lack of social interaction opportunities, significantly compromise welfare. To reinforce these standards, ABTA urges trade bodies and organizations to consistently monitor and verify that elephant-holding institutions adhere to the prescribed requirements for management and care.

#### 1.2.6 Guidelines on the Usage of Captive Elephants in Malaysia

The Malaysian Association of Zoological Parks and Aquaria (MAZPA) devised comprehensive guidelines specifically focused on captive elephants engaged in tourist activities across Malaysia (99). These guidelines cover a spectrum of practices, including performances, presentations, riding programs, and interactive sessions like feeding, photography, and bathing. MAZPA’s directives strictly prohibit physical threats or punitive measures toward elephants during these activities and emphasize the importance of conditions that mitigate unnatural behaviors. To ensure elephant comfort, the guidelines stipulate a minimum chain length of 4 meters with durations of less than 2 h between performances and housing on soft natural substrates Regular access to food and water is mandated, highlighting the crucial aspect of sustaining elephant health and vitality. Elephant handlers need to be qualified and knowledgeable in elephant care and using tools like the ankus and chaining.

## 2 Discussion

Within the two main welfare models used today, Five Freedoms and Five Domains, a range of methodologies exist for comprehensive welfare evaluations. To satisfy accreditation criteria, zoos and aquariums regularly evaluate the welfare of animals under their care (89), often using tools designed for multiple species. Each tool offers distinctive features that often serve different functions, such as complete behavioral and space utilization monitoring of ZooMonitor, global data sharing features of ZIMS, numerical and visual data representation of AWAG, or reliable and valid captive elephant measures presented by EBWAT. Tools range from digital formats to more traditional pen and paper for data recording and monitoring. However, overall, the trend is for institutions to use digital tools and advanced technologies to improve welfare standards (100). The tools differ in assessment levels, from assessments to understand individual variation to institutional level assessments that can inform on prioritization of resources and broadly benchmark progress in advancing welfare standards. These tools mostly rely on keeper ratings as a proxy for quantitative behavior assessments because keepers spend more time with the animals and can detect subtle changes that might be overlooked by others less familiar (81). Most also use a relative grading system; for example, AWRAP implements a 0–2 scale, Welfare Discussion Tool a 4-point scale, Ackonc-AWA a 3-point scale, and AWAG has a 10-point scale. Likert scales are also commonly utilized when evaluating behavioral indicators (25). ZIMS is flexible to allow users to select the grading in binary, numeric, and percentile values. Objective welfare scores allow the recording of welfare changes over time (25) and assist accreditation schemes in determining if an organization meets welfare requirements (67).

Across the tools, inputs range from observing behaviors to measures of health and stress indicators to provide comprehensive assessments across different welfare domains. Observational behavioral assessments emerged as a standard in all of the existing tools. Some tools use CCTV or cameras, while others rely on direct observations by keepers or other experts. In one study, ZooMonitor was used along with 18 closed–circuit cameras and five camera traps to record behavior states, habitat use, and social interactions of seven zoo Asian elephants (79). That study highlighted the benefits of combining ZooMonitor with other assessment methodologies for comprehensive welfare interpretations. Tools are increasingly using behavioral indicators associated with comfort, play, affiliation, foraging, and sociality to evaluate mental and overall welfare states, in addition to commonly used and validated negative welfare indicators like stereotypies, poor health and reproduction, and high mortality and morbidity (23, 44, 101, 102). To that end, the score sheet of WelfareTrak consists of positive (e.g., calm-relaxed) and negative (e.g., self-mutilating) behaviors. EBWAT includes stereotypies, social interactions, feeding, comfort, social behaviors, interactions with the environment, vocalizations, and others to measure mental health. AWAG also evaluates stereotypies, social affiliations, enrichment utilization, and responses to training as measures of psychological welfare.

Many tools also incorporate health evaluations as animal-based measures of physical condition. Stool and urine appearance, body coat condition, wounds, skin lesions, locomotion, micturition behaviors, general illness, teeth condition, and coat condition are all included in the health domain of Ackonc-AWA. Physical assessments in the AWAG include factors such as body condition scores, appetite, drinking and feeding behaviors, and activity levels, while the AWRAP tool includes body condition and an overall general health score, and the WDT overlays behavioral data with cortisol (feces, urine, etc.) analyses. In the case of elephants, cortisol or its metabolites can be measured in blood, saliva, urine, feces, and hair (103). Indeed, a study in India found zoos and sanctuaries with low welfare scores tended to have elephants with higher urinary cortisol and stereotypy rates (71). Immunoglobulin A (IgA) is among the novel biomarkers used as a positive welfare indicator and also in assessments of immune function (104). Like cortisol, IgA fluctuations can indicate positive and negative welfare states (105, 106) and be measured non-invasively. Combining analyses of glucocorticoids and IgA with behavioral indicators like stereotypies can further validate assessment findings (107, 108). Methods like allostatic load indexes are gaining attention because of their ability to capture cumulative stress (109), and so could potentially be used to predict mortality and morbidity risks. Other indices to consider could include evaluations of preference/avoidance, displacement, vocalization, startle/vigilance behaviors, salivary or urinary epinephrine, heart rate variability, and cardiovascular function.

Digital tools play a significant role in zoological institutions, enhancing efficacy, data visualization, and multi-institutional collaborations (81, 100). However, implementing these tools institutionally in non-zoological settings will be challenging. Elephant mahouts may have limited knowledge of technological devices to use ZooMonitor or WelfareTrak, and most camps do not have research staff or volunteers to input data. Thus, paper-based assessment methodologies might be more appropriate. It also can be challenging for low-budget venues in range countries to afford CCTV cameras and access to software to analyze data. In tourist camps, where elephants are engaged in activities like bathing, riding, and walking in natural forests (60), CCTV monitoring is impractical and could raise privacy concerns. Constantly updating information in digital tools like ZIMS could also be a logistical challenge for camp staff. Finally, most of these tools are only available in English, making them less useful for range countries.

The current reliance on keeper ratings or expert opinions in welfare assessment tools for captive elephants in range countries also has limitations. Although intimately familiar with their elephants, mahouts (i.e., elephant keepers) might not consistently identify stereotypic behaviors or have a comprehensive understanding of the full spectrum of elephant behaviors (50). Studies have highlighted instances where mahouts, despite their proximity to the animals, could not identify certain behaviors accurately, leading to discrepancies between direct observations and keeper assessments (92). Moreover, mahouts often face time constraints in non-zoological settings due to engaging in tourist interactions, impeding their ability to monitor behaviors continuously. The potential for positive bias in mahout ratings, influenced by personal attitudes and care for specific animals, also raises concerns about the objectivity of assessments (67). A more effective approach might involve a collaborative model that combines the expertise of mahouts and trained observers. This hybrid approach utilizes both perspectives synergistically, with mahouts offering unique insights into individual elephant social interactions and preferences. At the same time, trained observers conduct focused, objective behavioral assessments, especially when evaluating stereotypies.

Moving forward, there is a need to develop a new welfare assessment tool specific to elephants used in tourism. Tools should go beyond mere adaptability from zoo-centric models to incorporate components that address the specific dynamics, challenges, stressors, and ethical considerations found in tourist camps. The tool should integrate a balance of animal and resource-based measures and avoid the narrow focus on single behavior or health indicators (23, 55) to provide a comprehensive welfare risk assessment (67). With an increasing focus on using welfare assessment frameworks for developing assessment tools, the Five Domain Model can be adapted to develop the welfare assessment tool. Despite criticisms against the Five Domain Model (14), it is the most widely used model in animal welfare science and is important because of its focus on mental states. If limitations such as reliable and valid measures focusing on the overall mental and welfare state of captive elephants, and a structured welfare grading system are considered (14), the Five Domain Model can be adapted to develop a new welfare assessment tool. Previously established behavioral measures for captive elephants (23, 101, 102) and welfare factors associated with tourist camps can be integrated and adapted for further testing. The tool must be rapid, adaptable, undemanding in resources, non-invasive, and easy to complete, considering financial limitations, feasibility, and ethical concerns associated with invasive techniques (23, 25). Impractical measures like cognitive bias that require experimental setups (26) and measures such as safety from predators can be omitted, acknowledging their minimal impact on captive elephants in tourist camps. Despite recent efforts to enhance efficiency through technology (100), the practical constraints of tourist camps necessitate a focus on direct observation and questionnaires with mahouts. In the case of developing countries, a lack of understanding and awareness of animal welfare among mahouts makes it more challenging (94). To address this, a tool should integrate the perspectives of both mahout and experts, ensuring a more comprehensive and objective evaluation of elephant welfare. The tool should be designed to be executed by a trained individual familiar with the methodology, metrics, and relevant evaluation tools, intending to expand training to allow stakeholders and medical staff for in-house evaluation and assessments. Ensuring the tool’s validity, reliability, and practicality is paramount (23). Achieving validity involves integrating existing literature, expert consultancy, and adapting established and validated assessment measures (25). Reliability can be tested through inter-rater, reliability, repeatability, and internal consistency assessments. The tool should be able to track welfare changes over time, integrating objective and quantitative welfare scores. This integration facilitates the comparison of welfare levels for future evaluations, enabling institutions to meet accreditation. It provides a quantifiable means to interpret individual welfare states, reduce inter-observer variability, and the potential for intra- and inter-group comparisons to establish best practices in elephant welfare across diverse tourist camps. A range of factors, such as age, health status, reproductive status, and life history, need to be accounted for in welfare assessments of captive elephants. Animals of different ages may react differently to the same scenario or resource allocation (110). Having baseline data for specific age groups for later comparison will contribute to developing a credible tool (25). For example, in the U.S. most captive elephants have experienced at least one inter-zoo transfer (111), which is associated with stereotypic behaviors (35). Similarly, seasonality in cortisol or its metabolites is evident in African (112) and Asian (113) elephants and so must be considered when evaluating the physiological significance of fluctuations as stress indicators. For example, in Thailand tourist camp elephants, higher fecal glucocorticoid concentrations were observed during winter (November–February), presumably due to colder temperatures (49), but during an international travel ban in Thailand during the Covid-19 pandemic, the highest concentrations were in the rainy season, suggesting it is tourist activities that are the most likely cause of increased glucocorticoid excretion during the winter, high tourist season months (114).

Implementing tools developed by ZIMS (Care and Welfare module) and Wild Welfare might reveal issues that require costly improvements, conflicting with the profit-oriented approach of elephant tourism, making camps hesitant to adopt changes. Welfare concerns surrounding captive elephants in Asia encompass various activities such as the use of ankus, chaining, riding, performing in shows, logging work, training methods, weaning, participation in religious rituals or festivals, and even involvement in polo tournaments. These activities provoke international concern, but the upright dismissal of such practices could lead to tension between local communities and outside experts. Thus, establishing collaborations among all stakeholders is vital for informed management adaptations.

Addressing welfare challenges and implementing assessment methodologies also demands clear objectives, heightened awareness, robust legal frameworks, and collaborative endeavors involving governmental bodies (115). Organizations like the Asian Elephant Specialist Group (AsESG) (116), WAP, ABTA, and MAZPA are developing conservation action plans, guidelines, and manuals for elephants managed in range countries. However, governmental concerns are often overlooked. Thailand, for example, initiated efforts to improve elephant welfare in 2002 with welfare standards for elephant camps, later supplemented by additional standards in 2009 (64). However, compliance was low due to non-enforceability and limited incentives. Thailand passed the Cruelty Prevention and Welfare of Animals Act in 2014 to prevent cruelty and improve animal welfare, but it has yet to be implemented. The Asian Captive Elephant Standards (ACES) were created to promote the well-being of elephants in Southeast Asia but require sincere participation from elephant camps and strict welfare monitoring by governmental bodies (64). Hopefully, the elephant camp standards launched by the Thailand National Bureau of Agricultural Commodity and Food Standards implemented in August 2024 will bring positive changes regarding the welfare of elephants in tourist camps. Similar issues are evident in other regions, like India (56, 117, 118), where many captive elephants are kept in temples under dismal conditions. Unlike conventional zoo or sanctuary environments, these settings operate under distinct governance structures that are often less restrictive and more culturally influenced. Therefore, a tailored welfare assessment tool must navigate the delicate balance between traditional and modern welfare standards, recognizing the diversity of beliefs and practices surrounding captive elephant management.

## 3 Conclusion and future directions

Addressing the welfare concerns of elephants in non-zoological settings, particularly tourist camps, presents a pressing challenge. Existing animal welfare assessment tools, although flexible, often lack essential components for effectively monitoring and enhancing elephant welfare in these contexts. Many tools were initially designed for zoological settings, rendering them less practical for non-zoological environments. Digital tools and methodologies such as keeper ratings encounter difficulties when applied to tourist venues because mahout knowledge of elephant biology and behavior is more limited. Tools should consider critical factors like reliability, validity, practicality, and recent advances in animal welfare science for comprehensive assessments. By doing so, we can better identify welfare risks, inform management decisions, track welfare changes over time, and contribute to standardizing elephant welfare practices and policy-making processes in non-zoological settings. This review proposes that there is a need to develop holistic, context-specific, evidence-based, and practical assessment tools tailored to the unique needs of tourist camp elephants across Asia. Recognizing the limitations of current approaches, we are actively engaged in developing a novel assessment tool specifically designed for assessing the welfare of elephants in tourist camps. This initiative aims to fill the gaps identified in existing methodologies and promote higher welfare standards for elephants across Asian tourist venues. By employing a comprehensive and tailored approach, we aspire to foster positive welfare outcomes for elephants and contribute to the broader efforts aimed at enhancing animal welfare across diverse captive settings in Asia.

## Author contributions

RG: Conceptualization, Writing – original draft, Writing – review & editing. JB: Conceptualization, Writing – original draft, Writing – review & editing. CT: Conceptualization, Writing – original draft, Writing – review & editing. PB: Conceptualization, Writing – original draft, Writing – review & editing.
